# Tilt-evoked, breathing-driven blood pressure oscillations: Independence from baroreflex-sympathoneural function

**DOI:** 10.1007/s10286-024-01022-7

**Published:** 2024-03-06

**Authors:** Edward Grabov, Patti Sullivan, Siqi Wang, David S. Goldstein

**Affiliations:** 1 Autonomic Medicine Section, Clinical Neurosciences Program, Division of Intramural Research, National Institute of Neurological Disorders and Stroke, National Institutes of Health, 10 Center Drive MSC-1620, Building 10 Room 8N260, Bethesda, MD 20892-1620 USA; 2 Kentucky Spinal Cord Injury Research Center, University of Louisville, Louisville, KY USA

**Keywords:** Blood pressure, Baroreflex, Sympathetic, Power spectral analysis, Mayer wave

## Abstract

**Purpose:**

Orthostasis increases the variability of continuously recorded blood pressure (BP). Low-frequency (LF) BP oscillations (Mayer waves) in this setting are related to the vascular-sympathetic baroreflex. Mechanisms of increased high-frequency (HF) BP oscillations at the periodicity of respiration during orthostasis have received less research attention. A previously reported patient with post-neurosurgical orthostatic hypotension (OH) and vascular-sympathetic baroreflex failure had large tilt-evoked, breathing-driven BP oscillations, suggesting that such oscillations can occur independently of vascular-sympathetic baroreflex modulation. In the present study we assessed effects of orthostasis on BP variability in the frequency domain in patient cohorts with or without OH.

**Methods:**

Power spectral analysis of systolic BP variability was conducted on recordings from 73 research participants, 42 with neurogenic OH [13 pure autonomic failure, 14 Parkinson’s disease (PD) with OH, 12 parkinsonian multiple system atrophy, and 3 status post-brainstem neurosurgery] and 31 without OH (control group of 16 healthy volunteers and 15 patients with PD lacking OH), before, during, and after 5′ of head-up tilt at 90 degrees from horizontal. The data were log transformed for statistical testing.

**Results:**

Across all subjects, head-up tilting increased HF power of systolic BP variability (*p* = 0.001), without a difference between the neurogenic OH and control groups. LF power during orthostasis was higher in the control than in the OH groups (*p* = 0.009).

**Conclusions:**

The results of this observational cohort study confirm those based on our case report and lead us to propose that even in the setting of vascular-sympathetic baroreflex failure orthostasis increases HF power of BP variability.

**Supplementary Information:**

The online version contains supplementary material available at 10.1007/s10286-024-01022-7.

## Introduction

Orthostatic hypotension (OH) is defined by consensus as a persistent decrease in systolic blood pressure (BP) of ≥ 20 mmHg or in diastolic BP of ≥ 10 mmHg within 3 min of upright posture [7]. Assessing changes in BP and heart rate during head-up tilt table testing provides information about the reflexive changes in sympathetic noradrenergic and parasympathetic cardio-vagal drive in response to decreased venous return to the heart [3].

We recently reported the case of a patient with von Hippel-Lindau (VHL) disease and brainstem and cerebellar hemangioblastomas who developed disabling OH during convalescence after posterior fossa neurosurgery [13]. In this patient the OH was causally related to vascular-sympathetic baroreflex failure, as documented by abnormal BP responses to the Valsalva maneuver based on recordings using an automated finger cuff system [23] and by attenuation of the orthostatic increment in the plasma concentration of the sympathetic neurotransmitter norepinephrine [19]; that is, the patient had neurogenic OH [22]. Since the patient had extreme vascular-sympathetic baroreflex failure yet had intact cardio-vagal baroreflex function, the two efferent limbs of the baroreflex were affected differentially.

The patient had large tilt-evoked, breathing-driven oscillations of BP, while BP oscillations in the low-frequency (LF) range (0.15–0.5 Hz) were not present. The latter finding was expected, because LF BP oscillations (Mayer waves [9, 27]) are thought to result from resonance of central nervous and spinal zones regulating sympathetic noradrenergic outflows to the cardiovascular system [9]. In our patient this mechanism of LF oscillation was excluded, since he had profound vascular-sympathetic baroreflex failure.

Orthostasis can induce both LF and high-frequency (HF) BP oscillations. The latter correspond to the periodicity of respiration [4] and have been called Traube–Hering waves [1]. It has been shown that Traube–Hering waves persist through pharmacological approaches such as ganglionic blockade in healthy and autonomic failure cohorts [5, 38]. The findings in our patient, along with the above-cited research regarding Traube–Hering waves, led us to hypothesize that HF BP oscillations occur independently of baroreflexive modulation of sympathetic noradrenergic outflow, through purely mechanical effects.

We used a non-pharmacological approach to test our hypothesis, by analyzing physiological data from subject groups with vs. without neurogenic OH. We reviewed recordings from patients with OH who had physiological or neurochemical evidence of vascular-sympathetic baroreflex failure and in two control groups without OH. OH occurs in a substantial minority of patients with Parkinson’s disease (PD) [37], most patients with the parkinsonian form of multiple system atrophy (MSA-P), and all patients with pure autonomic failure (PAF).

For the purposes of the present study, PAF was defined by OH that had no identified secondary cause, was not associated with clinical signs of a movement disorder or cognitive dysfunction, and was associated with sympathetic noradrenergic deficiency as evidenced by low plasma levels of norepinephrine or its main neuronal metabolite 3,4-dihydroxyphenylglycol (DHPG) [14, 19]. PAF therefore was taken to include rare autoimmunity-associated forms of OH [autoimmune autonomic ganglionopathy (AAG) [17] and autoimmunity-associated autonomic failure with sympathetic denervation (AAD) [20]].

The results were compared with those in a control cohort of concurrently evaluated healthy volunteers (HVs) and patients with PD lacking OH (PD No OH) who underwent the same testing procedure.

## Methods

### Study subjects

All the participants in this study gave written informed consent before any research procedures were conducted. The protocol was approved by the institutional review board of the National Institute of Neurological Disorders and Stroke (NINDS) or the National Institutes of Health (NIH). All the patients had been referred for evaluation by the Autonomic Medicine Section (formerly Clinical Neurocardiology Section) of the Division of Intramural Research of the NINDS at the NIH Clinical Center.

All the patients had past medical records reviewed for signs and symptoms of autonomic dysfunction or a central movement disorder, underwent screening autonomic function testing at the NIH Clinical Center that confirmed the referral diagnosis [11, 29], and had additional clinical laboratory testing that supported and refined the findings from the screening examination. ^18^F-DOPA positron emission tomography (PET) was used to identify striatal dopamine deficiency [16], as typically occurs in patients with PD (with or without OH) and patients with MSA-P [16] but not in patients with PAF [18]. MSA-P was distinguished from PD+OH by ^18^F-dopamine PET [31]. In the VHL cohort, patients were recruited through consultation after neurosurgery for brainstem hemangioblastomas. In the control cohort the HVs had unremarkable medical histories and physical examinations and results of screening clinical laboratory tests done at the NIH Clinical Center within 6 months of research participation.

### Identification of neurogenic orthostatic hypotension

All the subjects in the neurogenic OH cohort satisfied criteria for vascular-sympathetic baroreflex failure on the basis of an algorithm published previously by our group [22]. Briefly, the patients had persistent, consistent OH without a known secondary cause such as medications or diabetes mellitus; had abnormal BP responses in both Phase II and Phase III/IV of the Valsalva maneuver [21, 23, 34]; had prolonged pressure recovery times after release of the maneuver [12, 34]; and in most cases had attenuated plasma norepinephrine responses to head-up tilt [19].

### Setup for autonomic function testing

The autonomic function testing was done in a dedicated Patient Observation Room operated by the Autonomic Medicine Section at the NIH Clinical Center. After urinating to empty the bladder, the participant lay supine with head on pillow on a motorized tilt table. An intravenous (IV) catheter was placed in an arm vein, usually the left antecubital vein, and attached by a 3-way stopcock and connection tubing to a plastic bag containing normal saline that was infused continuously at a slow rate to keep the vein open.

Physiological data were recorded using LabChart Pro 8 running a 16-channel PowerLab electronic physiological recorder (ADInstruments, Colorado Springs, CO). Electrocardiographic leads were attached to the skin and connected by a harness to the PowerLab. A noninvasive automated finger cuff system was used for continuous BP recording [BMEYE Nexfin, BMEYE B.V., Amsterdam, The Netherlands) or Finapres Nova (Finapres B.V., Amsterdam, the Netherlands)]. Respiration was monitored using a Respitrace plethysmography device placed around the upper abdomen or lower chest and connected to the PowerLab (ADInstruments, Colorado Springs, CO).

### Physiological tests

Physiological tests included slow, deep breathing at about 6 breaths per min, the Valsalva maneuver (30 mmHg for 12 s, ≥ 3 times until a technically adequate tracing was obtained [12, 21], with 20-degree head-up tilt in the event of a “flat top” BP pattern), and up to 5′ of head-up tilt at 90 degrees from horizontal. In the HVs head-up tilt could also be done at 70 degrees from horizontal for up to 40 min [3, 15].

### Blood sampling

Blood was drawn through the indwelling IV catheter into heparinized sample tubes during supine rest and at 5′ of head-up tilt (less than 5′ if the tilting was ended sooner for patient safety reasons). The plasma was separated by refrigerated centrifugation and transferred to plastic cryotubes for storage in an ultra-low temperature freezer. Freshly thawed samples were assayed for catechols by batch alumina extraction followed by liquid chromatography with series electrochemical detection as described previously [24].

### Power spectral analysis of blood pressure variability

Systolic BP data in the control, tilt, and recovery periods (Pre-tilt, Tilt, and Recovery) were analyzed using a cubic spline. Each segment was then linearly detrended. Power spectral densities of systolic BP were estimated using Welch’s method of averaged periodograms (300-point Hamming windows with 150-point overlap), using Matlab (MathWorks, Natick, MA). Spectral powers in the low-frequency (LF; 0.04–0.15 Hz) and high-frequency (HF; 0.15–0.4 Hz) bins were obtained using trapezoidal integration over the specified frequency range.

Physiological recordings were shortened during processing to undergo spectral analysis, and data were selected for the periods 5′ prior to tilt (Pre-tilt), 5′ during tilt (Tilt) if the patient withstood being upright for that long, and 5′ immediately upon return to horizontal (Recovery). In the few cases where patients did not tolerate 5′ of 90-degree tilt, a minimum of 1.5′ was used for power spectral analysis of the data during the tilt phase.

### Data analysis and statistics

GraphPad Prism 9 (GraphPad Software, LLC) was used for statistical analyses and graphics.

Cohorts were grouped on the basis of the diagnosis assigned for their disorder after comprehensive testing and the presence or absence of OH. Because of substantial interindividual variability that depended on the mean values, the power data were log transformed for statistical testing. Absolute differences in log-transformed data represent proportionate changes.

 Cardio-vagal baroreflex gain was calculated from the slope of the relationship between the cardiac interbeat interval (with 1-beat delay) and systolic BP between the highest BP value in Phase I and the lowest BP value during Phase II of the Valsalva maneuver [21].

The standard deviations (SD) of systolic BP in the Baseline, Tilt, and Recovery periods were used as measures of BP variability in the time domain.

Two-way analyses of variance (ANOVAs) were performed to compare groups across the Baseline, Tilt, and Recovery conditions using the tilt condition as the grouping factor for each cohort. Logarithmic transformations allowed for the data to meet necessary assumptions about homogeneity of variance for ANOVA testing. Repeated measures ANOVAs were used to examine differences among tilt conditions across individual groups. *F*-values were expressed as *F* (between groups df, within groups df), and effect sizes as partial eta squared. Dunnett’s post-hoc test was used for multiple comparisons of other cohorts vs. the HV group or Tilt vs. Baseline as appropriate. Mixed-effects analyses of power spectral data were carried out when there were missing data points using the same grouping factors as the two-way ANOVA.

To compare the control vs. neurogenic OH groups, independent means *t*-tests were used. To analyze changes from Pre-tilt to Tilt, pairwise comparison (dependent means) *t*-tests were used. A *p*-value less than 0.05 defined statistical significance.

## Results

Physiological recordings were reviewed from a total of 73 subjects, 43 in the neurogenic OH (nOH) and 21 in the control cohorts. The PD group consisted of 29 patients—14 in the PD+OH subgroup (10 males, 4 females, aged 68–89 years old) and 15 in the PD No OH subgroup (7 males, 8 females, aged 37–76 years old). The PAF group included 13 patients (8 males, 5 females, aged 60–85 years old), and the MSA-P group included 12 patients (8 males 4 females, aged 49–71 years old). The von Hippel–Lindau (VHL) brainstem neurosurgery group included 3 males, aged 25–48 years. The 16 participants in the HV cohort consisted of 10 males and 6 females, age range 29–63 years old. Among the cohort with nOH, the mean age was 67 years old (range 25–89 years old), and among the control cohort (HV and PD No OH) the mean age was 57 years old (range 29–79 years old; Supplementary Data Worksheet).

Average values for physiological data in the time domain are presented in Table 1.

**Table 1:** Mean (± SD) values for physiological variables in subject groups

Condition	HR*, bpm*	SBP, *mmHg*	Respiratory rate, *min*^*−1*^	Respiration frequency, *Hz*
Supine				
HV	66.8 (10.6)	128.1 (15.0)	18.2 (4.7)	0.30 (0.08)
PD No OH	66.9 (10.4)	138.4(23.2)	18.8 (6.4)	0.31 (0.11)
PD+OH	70.5 (11.9)	169.1 (37.2)	17.7 (4.4)	0.30 (0.07)
PAF	65.5 (9.1)	170.0 (36.1)	16.1 (3.4)	0.27 (0.06)
MSA	71.6 (8.3)	175.6 (45.7)	19.4 (4.8)	0.32 (0.08)
VHL	88.3 (18.3)	128.8 (26.0)	13.6 (6.4)	0.23 (0.11)
Tilt				
HV	79.1 (12.1)	142.5 (16.9)	17.4 (6.1)	0.29 (0.10)
PD No OH	75.0 (11.5)	147.3 (25.9)	17.0 (5.8)	0.28 (0.09)
PD+OH	79.7 (14.6)	129.9 (38.4)	17.0 (4.1)	0.28 (0.07)
PAF	73.5 (11.0)	119.1 (23.2)	16.5 (3.6)	0.27 (0.06)
MSA	82.5 (11.4)	129.6 (33.9)	16.3 (4.5)	0.27 (0.07)
VHL	109.4 (14.5)	97.4 (14.6)	14.5 (2.7)	0.24 (0.04)
Recovery				
HV	65.5 (11.6)	136.6 (15.5)	17.1 (4.9)	0.29 (0.08)
PD No OH	69.0 (13.9)	143.615 (29.9)	18.0 (6.6)	0.30 (0.11)
PD+OH	68.0 (10.7)	173.886 (29.4)	17.0 (3.8)	0.28 (0.06)
PAF	65.3 (8.1)	177.687 (27.2)	16.8 (3.4)	0.28 (0.06)
MSA	70.1 (8.8)	172.257 (46.3)	19.1 (4.8)	0.32 (0.08)
VHL	86.0 (21.3)	122.132 (12.8)	12.8 (6.3)	0.21 (0.10)

*HR* heart rate, *HV* healthy volunteer, *MSA* multiple system atrophy, *PAF* pure autonomic failure, *PD*+*OH* Parkinson’s disease with orthostatic hypotension, *PD* *No* *OH* Parkinson’s disease without orthostatic hypotension, *SBP* systolic blood pressure, *VHL* von Hippel-Lindau disease

### High-frequency power

Across all subjects, mixed-effects analysis showed that HF power varied across tilting conditions [*F* (1.552, 111.0) = 11.45, *p* < 0.001, partial eta squared = 0.1404, *df* = 72 for Baseline versus Tilt, and *df* = 71 for Baseline versus Recovery]. Based on Dunnett’s multiple comparisons test vs. the baseline condition, HF power increased between the Baseline and Tilt conditions (*p* = 0.002), while Recovery did not differ from Baseline (*p* = 0.180). Normalized HF power (HFnu) also varied as a function of tilting condition [*F* (1.960, 140.1) = 4.5, *p* = 0.010, partial eta squared = 0.0551, *df* = 72 for Baseline versus Tilt, and *df* = 71 for Baseline vs. Recovery], with both Tilt and Recovery having increased power from baseline (*p* = 0.020, *p* = 0.010). The patient groups did not differ from the HV group in HF power during any of the 3 conditions.

**Fig. 1:**
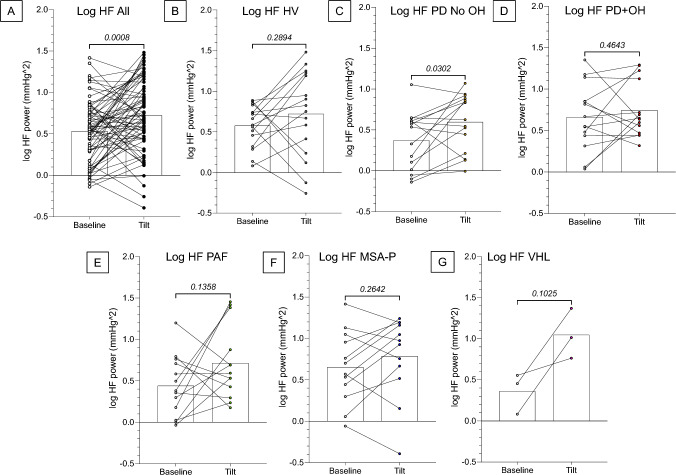
Individual values for the log of high-frequency (HF) power of systolic blood pressure (BP) variability before head-up tilt (Baseline) and during head-up tilt (Tilt) in different subject groups. **A** All study subjects; **B** healthy volunteers (HV); **C** Parkinson’s disease without orthostatic hypotension (PD No OH); **D** Parkinson’s disease with orthostatic hypotension (PD+OH); **E** pure autonomic failure (PAF); **F** parkinsonian form of multiple system atrophy (MSA-P); **G** von Hippel-Lindau disease with a history of neurosurgery for brainstem hemangioblastomas (VHL). Numbers in italics indicate *p*-values. Across all subjects, the log of HF power increased from Pre-tilt to Tilt. Within most groups, with the exception of PD No OH, there were no significant effects of tilt on HF power in any group

Comparing Tilt vs. Baseline by pairwise *t*-tests, head-up tilting increased the log of HF power across all subjects (*p* = 0.001, partial eta squared = 0.1457; Fig. 1A). Within all groups with the exception of PD No OH (*p* = 0.030, partial eta squared = 0.2934), there were no significant increases in the log of HF power between the Baseline and Tilt conditions (Fig. 1B–F).

### Low-frequency power

Across all subjects, LF power varied as a function of tilting condition [*F* (1.668, 119.3) = 4.6, *p* = 0.020, partial eta squared = 0.0618, *df* = 72 for Baseline vs. Tilt, and *df* = 71 for Baseline vs. Recovery]. Based on Dunnett’s post-hoc test, LF power during Tilt did not differ from that during Pre-tilt. Among HVs, LF varied with tilting condition [*F* (1.131, 16.96) = 4.1, *p* = 0.050], but neither Tilt nor Recovery differed from baseline. In the control cohort without OH (HV and PD No OH) mean LF power increased during tilt (*p* = 0.039), whereas patient groups with OH showed no significant changes.

Upon tilt, the HV group had significantly greater LF power than did the patient groups, which had highly variable responses and no significant overall changes. The HV group was statistically significantly different from all the patient groups (*p* = 0.002) and had higher LF power. On the basis of Dunnett’s post-hoc test comparing data in the patient groups with the HV group, statistically significant differences were found between HV and PAF (*p* = 0.002), PD+OH (*p* = 0.017), PD No OH (*p* = 0.016), MSA-P (*p* = 0.018), and VHL (*p* = 0.015).

**Fig. 2:**
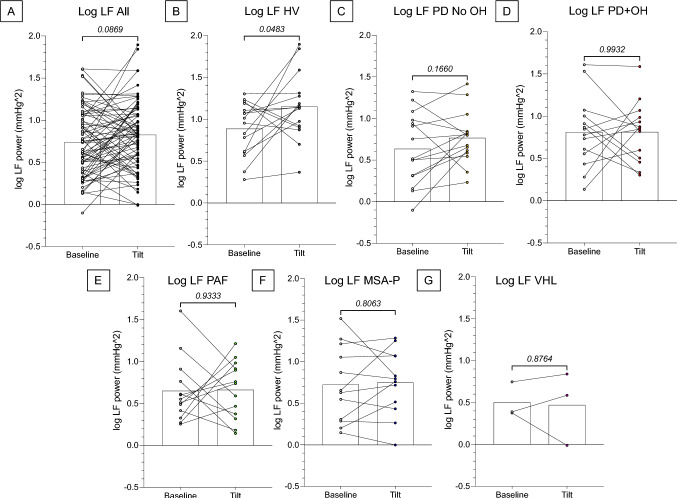
Individual values for the log of low-frequency (LF) power of systolic blood pressure (BP) variability during head-up tilt in different subject groups. **A** All the subjects in the study; **B** healthy volunteers (HV); **C** Parkinson’s disease without orthostatic hypotension (PD No OH); **D** Parkinson’s disease with orthostatic hypotension (PD+OH); **E** pure autonomic failure (PAF); **F** parkinsonian form of multiple system atrophy (MSA-P); **G** von Hippel-Lindau disease with a history of neurosurgery for brainstem hemangioblastomas (VHL). Numbers in italics indicate *p* values. The log of LF power increased significantly during tilt only in the HV group

Comparing Tilt vs. Baseline by pairwise *t*-tests, head-up tilting did not increase the log of LF power across all subjects (Fig. 2). The only group with a significant increase in the log of LF power during Tilt was the HV group (Fig. 2B).

### Control vs. Neurogenic OH cohorts

**Fig. 3:**
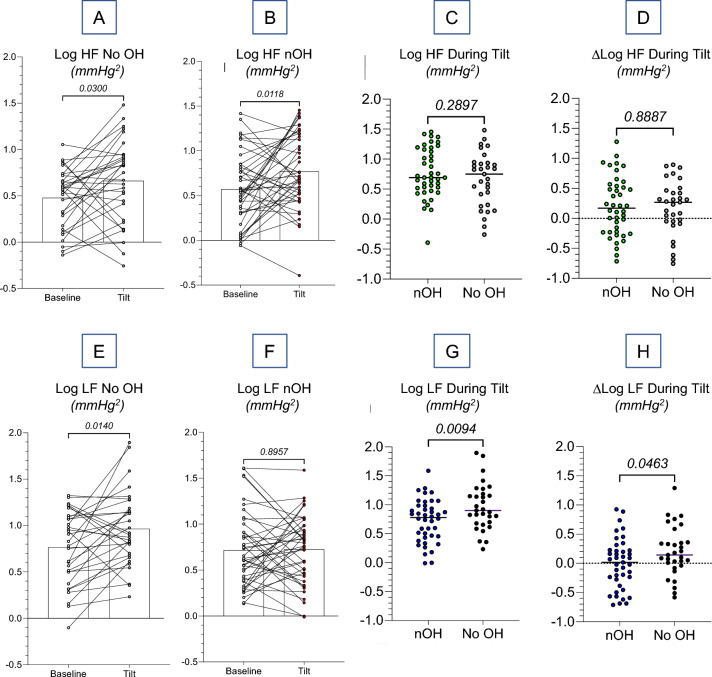
Individual values for the log of high-frequency (HF) and low-frequency (LF) power of systolic blood pressure variability before tilt (Baseline) and during tilt (Tilt) in groups without orthostatic hypotension (No OH) and with neurogenic orthostatic hypotension (nOH). The No OH cohort consisted of the healthy volunteer and Parkinson’s disease (PD) without OH groups. The nOH cohort consisted of the groups with pure autonomic failure, PD with OH, the parkinsonian form of multiple system atrophy, and von Hippel–Lindau disease with a history of neurosurgery for brainstem hemangioblastomas. Numbers in italics indicate *p*-values. As shown in **A**–**D**, the log of HF power increased with head-up tilt in both the control and nOH cohorts, and the two cohorts did not differ in the mean log of HF power during tilt or in the change in the log of HF power (∆Log HF) during tilt. As shown in **E**–**H**, the log of LF power increased with head-up tilt in the control but not in the nOH cohort. HF power in the nOH group is denoted by green circles (**C** and **D**) and LF power by blue (**G** and **H**)

In both the control and nOH cohorts the log of HF power increased between the Pre-tilt and Tilt conditions (Fig. 3A, B). The two cohorts did not differ in either the mean log of HF power during Tilt or the mean increment in the log of HF power from Pre-tilt to Tilt (Fig. 3C, D).

The log of LF power increased between the Pre-tilt and Tilt conditions in the control but not in the nOH cohort (Fig. 3E, F). The control cohort had a higher mean log of LF power during Tilt and a larger mean increment in the log of LF power from Pre-tilt to Tilt than did the nOH cohort (Fig. 3G, H).

The nOH cohort had lower mean cardio-vagal baroreflex gain (1.39 ms/mmHg) than did the control cohort (4.65 ms/mmHg, *t* = 5.658, *p* < 0.001, *df* = 59). Individual values for the log of HF power during Tilt were unrelated to the cardio-vagal baroreflex gain across all subjects (*r* = −0.01), in the nOH cohort (*r* = −0.13), and in the control cohort (*r* = 0.27).

In both the nOH and control cohorts SD BP increased between Pre-tilt and Tilt (for nOH, *t* = 3.26, *p* = 0.002; for control, *t* = 2.96, *p* = 0.006). Across all subjects, individual values for SD BP during Tilt tended to be negatively correlated with the log of the cardio-vagal baroreflex gain (*r* = −0.2337, *p* = 0.070). Pre-tilt SD BP was unrelated to the log of the cardio-vagal baroreflex gain.

### Examples of tilt-evoked blood pressure oscillations

**Fig. 4:**
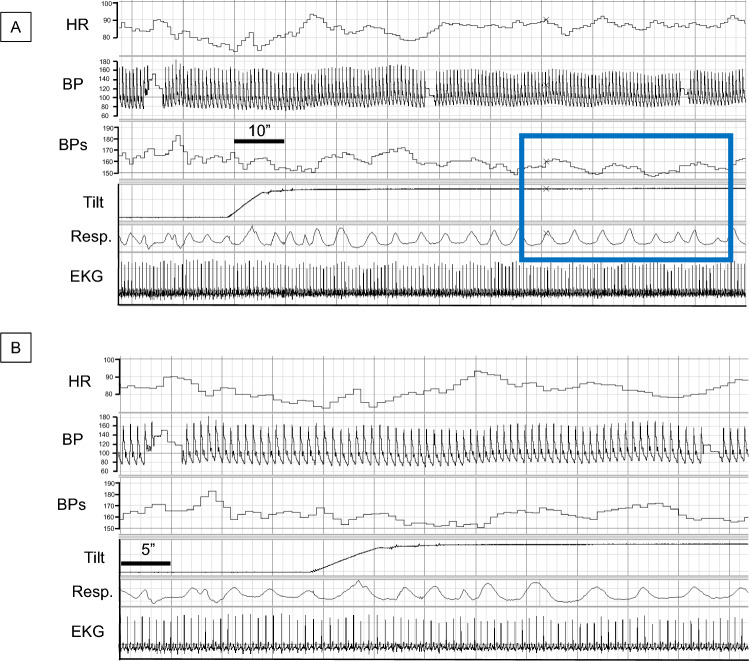
Low-frequency blood pressure (BP) oscillations during head-up tilting in a healthy volunteer. **A** and **B** are from the same recording but with different time scales. There is no orthostatic hypotension. During tilt, BP oscillations are not driven by breathing, and there are low-frequency Mayer waves. The blue rectangle is placed to highlight Mayer waves occurring at a lower frequency than that of respiration (Resp.). *BP* blood pressure (mm Hg), *BPs* systolic blood pressure (mmHg), *EKG* electrocardiogram, *HR* heart rate (bpm)

**Fig. 5:**
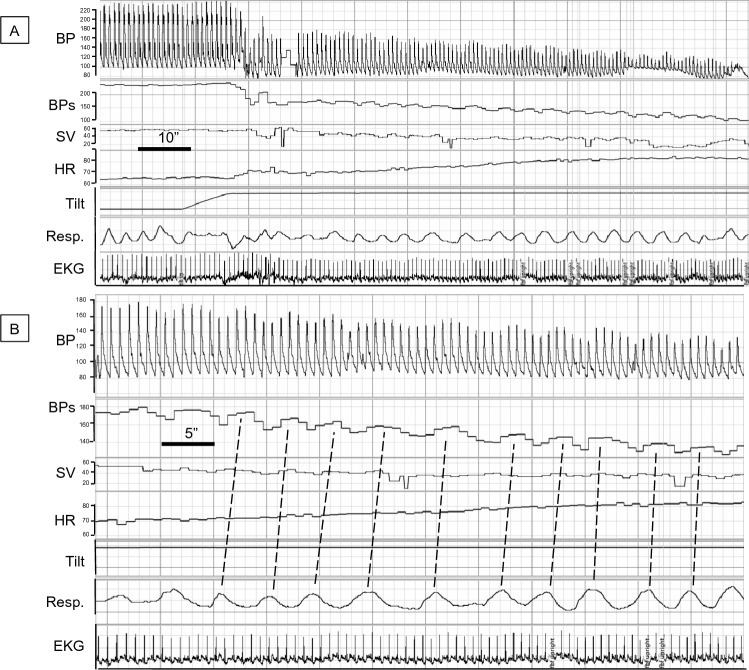
Tilt-evoked, breathing-driven blood pressure oscillations in a patient with neurogenic orthostatic hypotension (nOH) related to pure autonomic failure evolving to Parkinson’s disease with nOH. **A** and **B** are from the same recording but with different time scales. The patient develops rapid OH. During tilt, BP oscillations are driven by breathing. *BP* blood pressure (mm Hg) *BPs* systolic blood pressure (mmHg), *EKG* electrocardiogram, *HR* heart rate (bpm), *Resp*. respiration, *SV* stroke volume (mL)

**Fig. 6:**
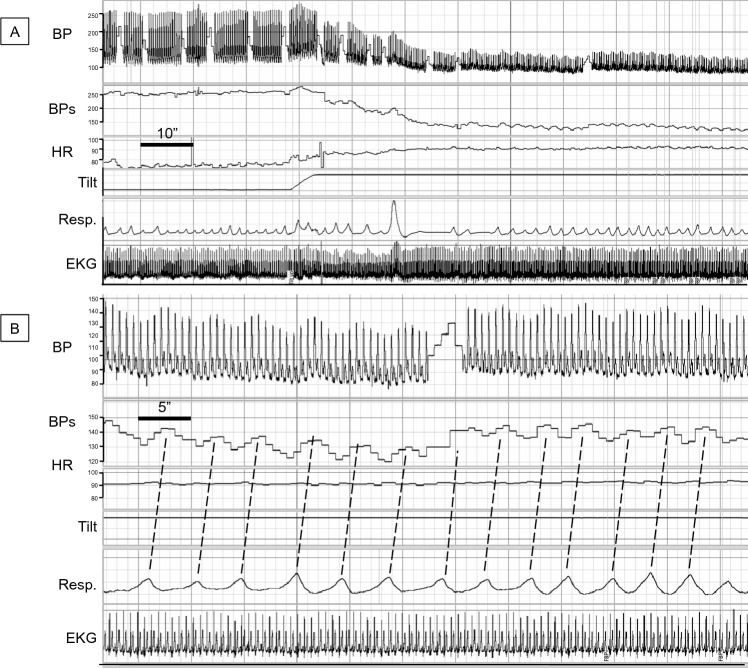
Tilt-evoked, breathing-driven blood pressure oscillations in a patient with neurogenic orthostatic hypotension (nOH) related to the parkinsonian form of multiple system atrophy. **A** and **B** are from the same recording but with different time scales. The patient develops rapid OH. During tilt, BP oscillations are driven by breathing. *BP* blood pressure (mm Hg), *BPs* systolic blood pressure (mmHg), *EKG* electrocardiogram, *HR* heart rate (bpm), *Resp.* respiration

Figure 4 shows a recording from a HV, and Figs. 5 and 6 show recordings from patients with nOH. In Fig. 4 the HV shows no evidence of breathing-driven BP oscillations, while low-frequency Mayer waves are evident. Figure 5 is a recording from a patient with PAF evolving to PD+OH, and Fig. 6 is from a patient with MSA-P. Both nOH patients have tilt-evoked, breathing-driven BP oscillations. It should be noted that such clear evidence was unusual, and many nOH patients did not have obvious increases in power of BP variability at the respiratory frequency during Tilt.

## Discussion

The impetus for the present study was an observation made during a clinical consultation in a patient with VHL who had disabling OH after neurosurgery for brainstem hemangioblastomas [13]. As expected, in this patient the OH was neurogenic (nOH), based on both physiological [12] and neurochemical [19] data. Unexpectedly, head-up tilt table testing evoked large BP oscillations at the relatively high frequency of breathing (Traube–Hering waves) [1]. Such oscillations were not present before or after the tilting. It seemed that tilt-induced HF BP oscillations might occur even in the setting of vascular-sympathetic baroreflex failure. This would be in contrast with relatively low-frequency Mayer waves, which complexly reflect baroreflex-mediated modulation of sympathetic cardiovascular outflows [4, 6, 25–28]. The purpose of the present study was to explore mechanisms of tilt-evoked, breathing-driven BP oscillations, by conducting power spectral analyses of systolic BP variability in patient groups with nOH (PAF, PD, and MSA-P) and a comparison cohort without OH (HVs and PD No OH).

The main new findings were that (1) across both the nOH and control cohorts HF power increased during tilting; (2) the cohorts did not differ in the magnitude of HF power during Tilt; and (3) the cohorts had a similar increase in mean HF power from Pre-tilt to Tilt. From these results we infer that tilt-evoked BP oscillations at the periodicity of breathing occur independently of vascular-sympathetic baroreflex modulation.

If Traube–Hering waves evoked by head-up tilting occurred independently of vascular-sympathetic baroreflex modulation, what would their mechanism be? The present results are inadequate to draw inferences on this point. Pharmacological blockade of ganglionic neurotransmission abolishes heart rate variability (the cardio-vagal component), yet Traube–Hering waves persist [2], and lung transplant patients who lack respiratory sinus arrhythmia still evince respiratory modulation of BP [36]. It has been proposed that breathing-driven increased BP variability occurs via two independent mechanisms—respiratory sinus arrhythmia and respiratory modulation of pulse pressure [1]. Until the present study, however, whether head-up tilting increases Traube–Hering waves in a manner independent of vascular-sympathetic baroreflex outflow had not been explored.

Baroreflexes contribute importantly to BP oscillations. Indeed, arterial baroreflex failure is always associated with decreased ability to buffer BP changes evoked by virtually any internal or external stimulus. Thus, patients with afferent baroreflex dysfunction as a late consequence of neck irradiation have highly variable BP during 24 h ambulatory monitoring [35], and ablation of the nucleus of the solitary tract, the site of initial synapses for baroreflexes, evokes chronic, labile hypertension in rats [33]; however, how these findings relate to tilt-evoked BP oscillations in the present study is unclear.

LF power of BP variability (corresponding to Mayer waves) was lower in the nOH than control cohort, as one would expect given that all the patients with nOH had vascular-sympathetic baroreflex dysfunction [8]. It has been proposed that Mayer waves are generated by brainstem and spinal cord inter-neuronal microcircuit oscillators that are likely modulated by baroreflexes [9, 10], although the exact mechanism in humans remains poorly understood.

A test of the hypothesis that tilt-evoked, breathing-driven BP oscillations can occur independently of vascular-sympathetic baroreflex modulation would be to evaluate individuals with chronic high spinal cord injury (SCI). Such patients typically have nOH owing to disruption of neurotransmission in the spinal cord. In this setting vascular-sympathetic baroreflex failure in SCI would be expected to occur without baroreflex cardio-vagal failure, since the vagus nerve exits the central nervous system from above the level of the SCI. Preliminarily, individuals with chronic SCI can have tilt-evoked Traube–Hering waves that resemble those in the nOH cohort in the present study (S. Wang, unpublished observations). Since spinal cord electrical stimulation can ameliorate the OH attending SCI [30], comparison of responses with vs. without spinal cord stimulation in individuals with SCI might provide a means to assess formally the roles of mechanical effects of respiration and of neural modulation on BP oscillations.

### Limitations

There was marked individual variability in responses of both HF and LF power to tilting in all the groups in this study, and the durations of recording periods were relatively short, further limiting data reliability.

If tilt-evoked Traube–Hering waves were purely mechanical, respiratory amplitude and venous return to the heart would be expected to play important roles in the BP oscillations, but neither variable was tracked. Individuals probably vary in breathing responses to tilting, yet neither the rate nor depth of respiration was controlled. It was evident that some patients had slower breathing during tilting than at Baseline or during Recovery, meaning that part of the breathing-driven BP oscillations during tilting were in the LF range. Effects of orthostatic changes on respiratory frequency would have influenced the variability of power data calculated from within predefined frequency bins. In future studies on this topic individual variability might be reduced by having subjects perform paced breathing during orthostasis. There may also have been large individual differences in the effects of orthostasis on venous return to the heart and consequently on cardiac stroke volume and pulse pressure. Although the automated finger cuff systems we used for tracking BP continuously came with software applications that reported estimated values for beat-to-beat stroke volume, to our knowledge the algorithms have not been validated in nOH. Another possible complicating factor is the chemoreflex [32], but capnography was not performed.

## Implications and conclusions

The results of this retrospective observational study confirm those based on our case report describing tilt-evoked, breathing-driven BP oscillations in a patient with post-neurosurgical vascular-sympathetic baroreflex failure. In general, our results support the view that HF BP oscillations during orthostasis are mainly, if not purely, mechanical. Based on the present results it might be informative to assess whether large tilt-evoked, breathing-driven BP oscillations precede tilt-evoked sudden hypotension in patients with chronic orthostatic intolerance.

### Supplementary Information

Below is the link to the electronic Supplementary Material.

Supplementary File 1. Individual demographic and physiological data about tilt-evoked blood pressure oscillations. Abbreviations and Data Dictionary: *BP* systolic blood pressure, *HF* high-frequency power of systolic blood pressure variability, *HV* healthy volunteers, *Log HF* log of high-frequency power of systolic blood pressure variability, *∆Log HF* change in Log HF from Pre-tilt to Tilt, *LF* log of low-frequency power of systolic blood pressure variability, *MSA-P* parkinsonian multiple system atrophy, *PAF* pure autonomic failure, *PD+OH* Parkinson’s disease with orthostatic hypotension, *PD control* Parkinson’s disease without orthostatic hypotension, *Pre-tilt* supine rest before head-up tilting, *Recov*. supine rest after head-up tilting, *SD* standard deviation of systolic blood pressure, *∆SD* change in SD from Pre-tilt to Tilt, *Tilt* head-up tilt, *VHL* von Hippel–Lindau disease after neurosurgery for brainstem hemangioblastoma (XLSX 28 KB)
